# Alzheimer's disease: an epidemiological analysis over the number of hospitalizations and deaths in Brazil

**DOI:** 10.1055/s-0043-1767827

**Published:** 2023-06-28

**Authors:** Eduardo Cattapan Piovesan, Bruna Zanatta de Freitas, Francisco Costa Beber Lemanski, Charles André Carazzo

**Affiliations:** 1 Universidade de Passo Fundo, Faculdade de Medicina, Passo Fundo RS, Brazil.; 2 Instituto de Neurologia e Neurocirurgia de Passo Fundo, Passo Fundo RS, Brazil.

**Keywords:** Dementia, Alzheimer Disease, Epidemiology, Unified Health System, Brazil, Demência, Doença de Alzheimer, Epidemiologia, Sistema Único de Saúde, Brasil

## Abstract

**Background** Alzheimer's disease (AD) is a neurodegenerative condition characterized by impaired cognitive function. It results in high morbidity, including a large number of hospitalizations, and mortality, generating high costs to health systems.

**Objective** The present epidemiological analysis evaluated the number of hospitalizations and deaths by AD as the main diagnosis in Brazil between 2010 and 2020. This endeavor should contribute to a better understanding of the disease and its implications.

**Methods** The present analytical, observational, longitudinal, and retrospective study used data extracted from the Department of Informatics of the Brazilian Unified Health System (DATASUS, in the Portuguese acronym). The variables include the number of hospitalizations, the total cost spent, the average cost per hospitalization, the average length of hospital stay, the number of deaths during hospitalization, the mortality rate per hospitalization, sex, age group, region, and race.

**Results** From 2010 to 2020, there were 188,811 deaths and 13,882 hospitalizations for AD, with a total expenditure of BRL 25,953,019.40 in hospitalizations. The average length of hospital stay was 25 days. Over the considered period, mortality, the number of hospitalizations, and the total cost increased while the average length of stay decreased.

**Conclusion** From 2010 to 2020, AD represented a large portion of hospital admissions, generating a significant cost to the health system and a large number of deaths. These data are important to undertake joint efforts to prevent hospitalizations of these patients in order to minimize impacts on the health system.

## Resumo

**Antecedentes** A doença de Alzheimer (DA) é uma doença neurodegenerativa caracterizada pelo comprometimento da função cognitiva. Resulta em uma alta taxa de morbimortalidade, por meio de um número significativo de óbitos e internações, gerando um alto custo ao sistema de saúde.

**Objetivo** Realizar uma análise epidemiológica, utilizando as variáveis citadas abaixo, por DA como principal diagnóstico, no Brasil, entre 2010 e 2020, a fim de contribuir para um melhor entendimento da doença e suas implicações.

**Métodos** Estudo analítico, observacional, longitudinal e retrospectivo. Os dados foram extraídos do Departamento de Informática do Sistema Único de Saúde (DATASUS). As variáveis utilizadas foram número de internações, custo total gasto, custo médio por internação, tempo médio de internação, número de óbitos nas internações, taxa de mortalidade por internação, número de óbitos absolutos, sexo, faixa etária, região e raça.

**Resultados** De 2010 a 2020, foram registrados 188.811 óbitos e 13.882 internações por DA, com um gasto total de R$ 25.953.019,40 em internações. O tempo médio de permanência no hospital foi de 25 dias. Em 11 anos, houve aumento da mortalidade, internações e custo total; por outro lado, o tempo médio de permanência, no mesmo período, diminuiu.

**Conclusão** A DA, nos anos avaliados, representa uma parcela significativa das internações hospitalares, gerando um custo significativo ao sistema de saúde, além do número de óbitos. Estes dados são importantes para gerar esforços conjuntos para evitar internações desses pacientes, a fim de minimizar o impacto no Sistema de Saúde.

## INTRODUCTION

Alzheimer's disease (AD) is a neurodegenerative condition characterized by an insidious onset and gradual development of cognitive dysfunction, with an impact on common daily functions and the development of neuropsychiatric symptoms. 1 2 It was first described in 1907 by Aloysius Alzheimer, who microscopically evaluated the brain of a woman who presented with delusional symptoms, language and memory changes, and spatiotemporal disorientation. He observed neuritic plaques, neurofibrillary tangles, and amyloid angiopathy, which have become the hallmarks of the disease. 3 4 5

AD is a complex disorder that is responsible for almost three quarters of all cases of dementia. 6 7 In early AD, the impairment is mild, and the most frequent symptoms are memory deficit, depression, and apathy. In advanced stages, the memory deficit is exacerbated, and symptoms such as delusions, hallucinations, and aggressiveness become common. Finally, with progression, the patient can become unresponsive. 8 9

Life expectancy has increased throughout the world; a consequence of this change is that more people are affected by degenerative diseases, including AD. 2 10 Globally, the number of people affected by dementia was estimated to have increased by 117% between 1990 and 2016, largely due to population aging. 11 In the United States, epidemiological data show that AD affects > 5.5 million people, and other research indicates that it affects ∼ 24 million people worldwide. 12 It is estimated that dementia will affect more than 81 million people by 2040. 4 In 2016, Brazil had the second highest age-standardized prevalence of dementia in the world, affecting ∼ 1.7 million people, and projections indicate that the average prevalence is higher than the global prevalence. 13 14 A likely consequence of this would be an increase in hospital admissions as well as in general costs and direct or indirect deaths.

According to the Alzheimer's Association, people with AD and other forms of dementia are hospitalized twice as much per year as other older people without dementia. 15 Lin et al. 16 stated that hospital admissions represent the largest component of health care expenditures for individuals with AD and related disorders and constitute more than half of total expenditures among patients with prominent comorbidities. In the United States, ∼ 40% of all annual hospitalizations from 2000 to 2008 were people ≥ 85 years old living with dementia. 14 From 2010 to 2019, AD had the highest increase in number and rate of hospitalizations among the top 5 causes of death by noncommunicable disease in Brazil. 14 According to the Brazilian Ministry of Health, the number of hospitalizations due to AD increased 88% from 2010 to 2019. For comparison purposes, hospitalizations due to cerebrovascular and ischemic heart disease increased 36.3 and 29.3%, respectively, during the same period. 17

AD is listed as the sixth leading cause of death in the United States for those ≥ 65 years old. According to data from the National Center for Health Statistics of the Centers for Disease Control and Prevention (CDC), in 2013, 84,767 people in the United States died from AD. 15 In 2018, > 122,000 people died from AD, an increase of 146% from the year 2000. 18 In Brazil, from 2007 to 2017 the number of deaths due to dementia increased by 55.5%, a greater increase than breast, prostate, and liver cancer deaths combined. 17 In Argentina, Chile, and Uruguay, the number of deaths attributable to dementia has decreased by up to 5.3%, but the mortality rate in Brazil increased by 12.5% per year from 2000 to 2008. 14 The high prevalence and mortality rate have increased the burden of AD on the Brazilian hospital system.

“If dementia care were a country, it would be the world's 18th largest economy.” This statement from the Alzheimer's Association highlights the magnitude of the economic burden of dementia, which will increase dramatically in the near future. 19 20 The costs of healthcare, long-term care, and hospice for individuals with AD and other forms of dementia are substantial, and AD is one of the costliest chronic diseases to society. In 2016, total payments for all individuals with AD and other form of dementia were estimated at US$236 billion. 15 In 1998, Meek et al. 21 stated that in terms of total costs to society, AD was the most expensive disease in the United States, after cancer and coronary heart disease. 21 According to the Brazilian Ministry of Health, the total economic cost due to AD increased 44% from 2010 to 2019. The annual total cost per individual living with dementia in Brazil is greater than the global average regardless of disease stage. 14 In this context, and because there are few studies on the subject, the present epidemiological study between the years 2010 and 2020 evaluates the number of hospitalizations and deaths due to AD in Brazil.

## METHODS

An analytical, observational, longitudinal, and retrospective study was conducted analyzing epidemiological data regarding AD – as the main diagnosis and the cause of hospitalization and death – in Brazil between 2010 and 2020. The data were obtained from the Hospital Information System (SIH, in the Portuguese acronym) and the Mortality Information System (SIM, in the Portuguese acronym) collected by the Department of Informatics of the Brazilian Unified Health System (DATASUS, in the Portuguese acronym) through the TabNet tool. 22 The variables used were the number of hospitalizations, the average length of hospital stay, the total cost spent, the average cost per hospitalization, the number of deaths in hospitalizations, the mortality rate per hospitalization, and the number of absolute deaths.

The present study considered all variables from which the primary diagnosis was AD, defined as G30.0–G30.9 according to the International Classification of Disease, 10th revision (ICD-10). 23

The mortality rate per hospitalization is the ratio of the number of hospitalized patients with AD who died to the number of hospitalized patients with a primary diagnosis of AD in the corresponding period.

The number of hospitalizations and the number of absolute deaths were stratified into sex (male/female), region (North, Northeast, Southeast, South, and Center-West), age groups (< 50, 50–59, 60–69, 70–79, and > 80 years old), and race (white, black, brown).

The data were tabulated and later converted into graphs using Microsoft Excel 2007 software (Microsoft Corporation, Redmond, WA, USA). For descriptive analysis, the proportional means and percentages of each indicator were calculated.

There was no need to obtain Research Ethics Committee approval because all analyses were based on the use of secondary data, without identification of participants, as determined by Resolution No. 466 of the National Health Council of December 12, 2012.

## RESULTS

**Figure 1:**
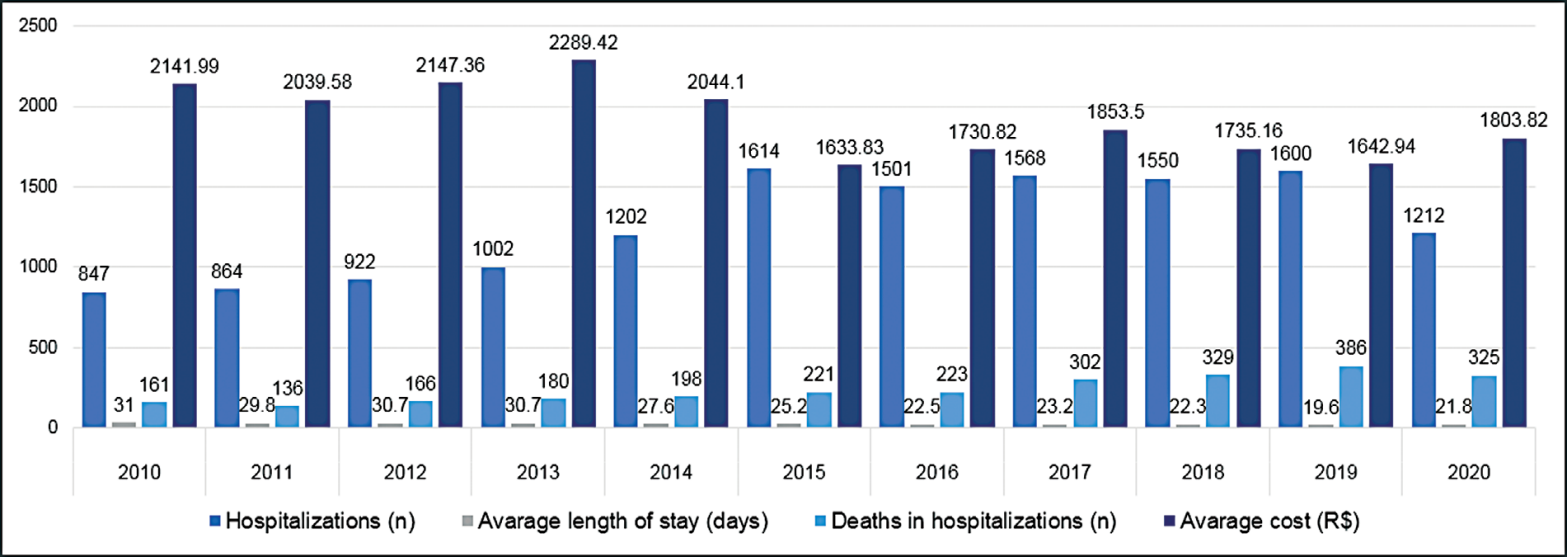
Data on hospitalizations for Alzheimer's disease, 2010–2020.

From 2010 to 2020, there were 13,882 hospitalizations due to AD, with an average length of stay of 25 days. The number of hospitalizations increased over the years, from 847 in 2010 to 1,212 in 2020. However, at the same time that the number of hospitalizations increased, the average length of stay decreased. In 2010, the average was 31 days, while in 2020 the average was 21.8 days ( Figure 1 ).

**Figure 2:**
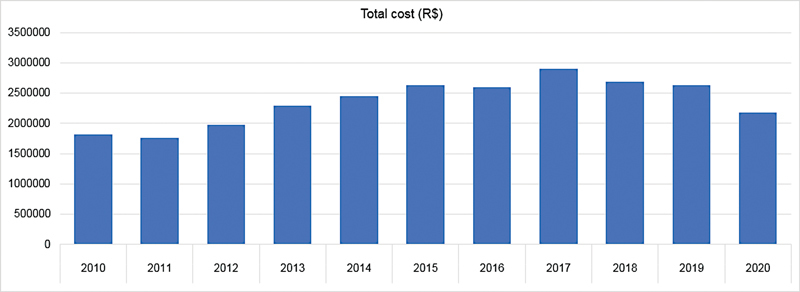
Total cost of hospitalizations for Alzheimer's disease, 2010–2020.

Regarding the total cost, BRL 25,953,019.40 was spent on hospitalizations for AD in Brazil. The average cost per hospitalization in the aforementioned period was BRL 1,869,54. The amount spent on AD increased over the considered period, while the average cost decreased. In 2010, the total cost was BRL 1,814,265.94, with an average cost per hospitalization of BRL 2,141.99. In 2020, the values were BRL 2,186,228.84 and BRL 1,803.82, respectively ( Figures 1 and 2 ).

**Figure 3:**
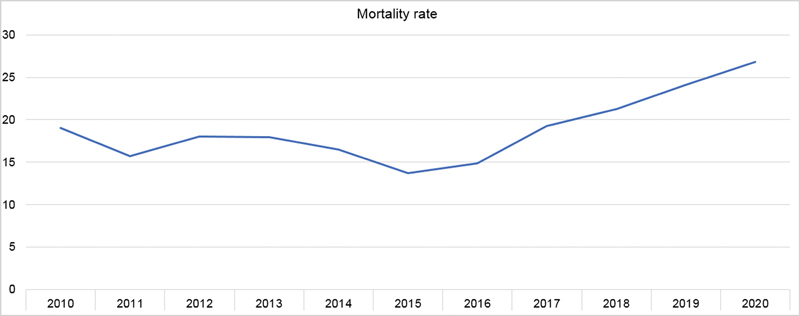
Mortality rate in patients hospitalized for Alzheimer's disease, 2010–2020.

From 2010 to 2020, 2,627 patients with AD died in the hospital. Like the number of hospitalizations, this number increased over the considered period. In 2010, there were 161 deaths from 847 hospitalizations. In 2020, there were 325 deaths from 1,212 hospitalizations. Over the considered period, there was a 101.96% increase in the number of patient deaths ( Figure 1 ). The average mortality rate in hospitalized patients in the analyzed period corresponds to 18.92%, and a significant increase from 2016 (14.6%), a period in which it began to increase and evolved until reaching its maximum in 2020 (26.82%) ( Figure 3 ).

**Figure 4:**
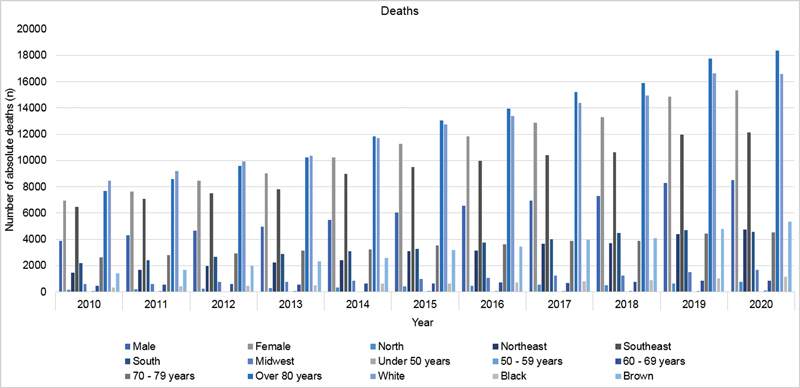
Data on absolute mortality from Alzheimer's disease, 2010–2020.

The number of deaths from AD from 2010 to 2020 was 188,831. There was an increase in the number of deaths per year during this period, from 10,841 deaths in 2010 to 23,855 deaths in 2020, an increase of 13,014 deaths ( Figure 4 ).

The total number of deaths from AD was highest in the Southeast (54.25%) and the South (20.15%), followed by the Northeast (17.16%). The region with the lowest number of deaths was the North, with 4,579 (2.42%). Moreover, the Midwest region accounted for 6% of deaths. Of the absolute number of deaths, there was a predominance of females (64.52%, 121,821 deaths) compared with males (35.48%, 66,990 deaths). Regarding age, the highest number of deaths occurred in individuals > 80 years old (75.25%), followed by 70 to 79 years old (20.42%) and 60 to 69 years old (3.84%). The age group with the lowest mortality was individuals < 50 years old, with 126 recorded deaths. Individuals aged 50 to 59 years old represented 0.42% of total deaths. Most of the people who died from AD were white (73.27%), followed by brown (18.42%) and black (4%). From 2010 to 2020, there was a significant increase in the number of deaths for the various analyzed factors. As with the total number of hospitalizations, there is a greater risk in the number of deaths as age increases, so that individuals aged from 70 to 79 years old and > 80 years old represent almost all the deaths from AD. Moreover, most deaths occurred in patients living in the Southeast and South regions ( Figure 4 ).

**Figure 5:**
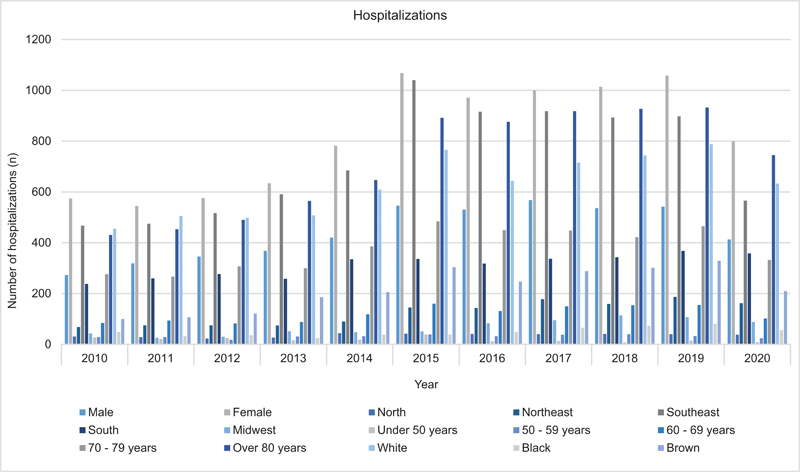
Data on hospitalizations for Alzheimer's disease, 2010–2020.

The Southeast (57.38%) and South (24.69%) regions had the highest number of hospitalizations, the North region (2.84%) had the fewest, and the Northeast (9.77%) and Midwest (5.3%) regions were in between. Of the total number of hospitalizations, there was a predominance of women (64.8%, 9,021 hospitalizations) compared with men (35%, 4,861 hospitalizations). The age group with the highest number of hospitalizations was > 80 years old, accounting for 56.7% of the total, followed by 70 to 79 years old (29.8%) and 60 to 69 years old (9.49%). The age group with the fewest hospitalizations was < 50 years old (1.48%). Finally, individuals aged from 50 to 59 years old accounted for 2.48% of hospitalizations. The white race had the highest number of hospitalizations (68.9%), followed by the brown race (24%) and the black race (5.5%). The number of hospitalizations increased from 2010 to 2020 for each age group, except for individuals < 50 years old and from 50 to 59 years old. In these groups, the number of hospitalizations decreased over the considered period, from 27 and 29, respectively, in 2010, to 9 and 24, respectively, in 2020 ( Figure 5 ).

## DISCUSSION

The recent statistical data about the epidemiology of AD in Brazil are worrisome. Feter et al. 14 reported that, from 2010 to 2019, there was an increase in hospitalization attributable to AD, and it was higher than hospitalization for cerebrovascular and ischemic heart diseases. Similarly, the increase in costs associated with AD care in hospital settings was higher than chronic obstructive pulmonary disease and diabetes mellitus. In 2020, there was a 23.7% reduction in the number of hospitalizations, and a 3% increase in the number of deaths compared with 2019 ( Figures 1 and 4 ). Despite the reduction in hospitalization, the numbers remain high. The data can be justified by the COVID-19 pandemic because the disease is a risk factor for AD mortality and delayed hospitalization for several diseases.

The findings from the present study indicate a significant increase in the number of hospitalizations, the total cost spent, the average cost per hospitalization, the number of deaths during hospitalization, the mortality rate per hospitalization, and the number of deaths. Thus, AD has a profound effect on society denoted by the high morbidity and mortality it causes. Similarly to changes throughout the world, in Brazil the absolute mortality due to AD has increased. From 2010 to 2020, the absolute number of deaths increased by 120% ( Figure 4 ). The number of hospitalizations, as well as the absolute number of deaths, increased 40% ( Figure 1 ). Moreover, the total costs increased by 20% ( Figure 2 ). However, the average cost per hospitalization decreased by 16%, because the average time per hospitalization decreased ( Figure 1 ). A reduction in the hospital length of stay can be justified by the earlier discharge of patients with AD or because the patients are dying earlier than expected. Moreover, the increased number of hospitalizations and deaths can be justified by the increased number of AD diagnoses.

Similarly to the increase in the total cost of hospitalization in the Brazilian Unified Health System, there has been a global increase in spending on AD. In 1998, in terms of total costs, it was the third most expensive disease in the United States, and in the 2000s, it is estimated that the annual expenditure approached US$100 billion. 13 24 In China, the total socioeconomic costs of patients with AD were estimated to be US$167.74 billion in the year 2015. 25 Research indicates that AD costs the world US$1 trillion, and this figure is expected to double in the next 30 years. 14 The situation is not different in Brazil. According to Feter et al., 14 from 2010 to 2019, the economic cost of hospitalizations for AD increased 41.6%. For the Brazilian Unified Health System, data regarding drug treatment indicate that there was a 109% increase in expenses over a 5-year period, from R$75.6 million in 2007 to R$157.8 million in 2011. 26 27

Based on prior studies and the population aging, the number of deaths and hospitalizations due to AD in Brazil has increased. Thus, epidemiological knowledge about each of these factors is crucial. Although great advances have been made in AD research, little attention has been paid to sex differences. In the present study, there were more deaths and hospitalizations in women compared with men. Indeed, 65% of hospitalizations and 64.5% of deaths occurred in women. Nevertheless, men presented a greater increase in hospitalization compared with women: a 51% increase from 2010 to 2020, compared with a 39% increase in females. Alzheimer's disease was more prevalent in white individuals: They accounted for 68.3% of the total number of hospitalizations and 73.27% of the total number of deaths.

The greater prevalence of AD in women found in the present study is consistent with other epidemiological research. The female sex is a major risk factor for developing AD. It is estimated that of the 5.3 million people aged ≥ 65 years old with AD in the United States, 3.3 million are women and 2.0 million are men. The estimated lifetime risk of AD at 45 years old is ∼ 1 in 5 for women and 1 in 10 for men. 28 29 The higher incidence in females may be because women live longer than men and, after menopause or over the age of 60 years old, women have a higher prevalence of obesity, diabetes, and other conditions that increase the likelihood of developing AD. 30 31

In contrast to the present study regarding the higher prevalence of hospitalization and death from AD in white individuals, global statistics indicate a higher prevalence in black individuals. Rajan et al. 32 found that the prevalence of AD was higher among African American individuals compared with white individuals. This finding is consistent with previous findings that the prevalence of AD is much higher in African Americans than in other racial and ethnic groups. Rubin et al. 33 indicated that African American individuals are twice as likely as non-Hispanic White individuals to have AD, while Hispanic individuals are 1.5 times more likely to have AD compared with non-Hispanic white individuals. Death by cerebrovascular disease, hypertension, and diabetes has contributed to the “white–black” gap in life expectancy in Brazil, with black and mixed ethnicity individuals dying earlier due to those chronic conditions. 14 Research indicates that racial inequalities in life expectancy and access to health care in Brazil may explain, in part, the lower likelihood of AD among black individuals. The 2020 Lancet Commission report showed that racial disparity is still a factor to be addressed in the management of AD and other forms of dementia. Equal access to health care facilities is one way to reduce racial inequality in the burden of AD and other forms of dementia. 17 34

Alzheimer's disease was more prevalent in the South and Southeast regions, which together represented almost all hospitalizations and deaths. However, the North and Northeast regions showed the greatest increases in death, with a 363.6 and 227.4% increase, respectively, from 2010 to 2020 ( Figure 4 ). The number of hospitalizations increased the most in the Northeast (138.2%) and the Midwest (104.7%) regions ( Figure 5 ). The geographical distribution of mortality and hospitalizations showed a greater increase in the North, Northeast, and Central regions. This pattern can be explained by improved access to diagnostics, a phenomenon that has been observed with other chronic diseases. The Southeast and South regions are significant economic and commercial centers in Brazil, besides being the most economically developed regions in the country. Thus, they offer more health programs and services, trained health care workers, and technological resources to the population. 35

Age is directly proportional to mortality and the number of hospitalizations due to AD. In the present study, individuals > 70 years old represented almost all deaths and hospitalizations ( Figures 4 and 5 ). This finding is consistent with a comparative epidemiological study that showed that age is the most important risk factor for the development and severity of AD. The authors analyzed 1,246 individuals aged from 30 to 95 years old and found that AD risk increased with age, particularly after 70 years old. 36 Another study showed that the prevalence of AD approximately doubles every 5 years in individuals aged from 65 to 85 years old: from ∼ 1 to 2% at age 65, to > 30 to 50% at age 85. 37 In a population-based study conducted in Brazil, the authors observed a dementia prevalence of 7.1% (118 inhabitants) in a population of 1,656 individuals aged ≥ 65 years old. Of these, 55.1% of the dementia cases were diagnosed as AD. 24 According to the Brazilian Institute of Geography and Statistics (IBGE, in the Portuguese acronym), the population ≥ 60 years old will increase by 284.2% from 2000 to 2050, and the probability of an AD medical diagnosis among the sample increases by 11% for each year of increased aging. 17

The data from the present study have been influenced by the COVID-19 pandemic. There has been a reduction in the number of hospitalizations for diseases other than COVID-19. In addition, AD affects older patients and, for this reason, is associated with other comorbidities. Hence, the status of the population studied in the is a risk factor for mortality associated with COVID-19.

The present study has several limitations. First, because it involves data obtained from a public database, confounding factors could not be controlled and reliability in the processing of the information used could not be guaranteed. Another limitation is the fact that the data do not specify the severity of the patients who were hospitalized, nor the reasons, which may compromise the results on mortality. Other limitations are that, through DATASUS, the present study cannot provide information like a patient's nutritional status, risk factors, multimorbidity, and fragility, which interferes with determining the number of deaths and the average hospital length of stay.

In conclusion, the present epidemiological study contributes significantly to understanding AD and its implications, and indirectly serves to verify whether public health policies minimize the damage caused by AD as well as the costs to the state. Effective public policy can be created when its formulators are familiar with the epidemiology associated with a particular disease. Considering that AD is complex, its development involves multiple factors, and epidemiological studies on AD are limited; knowledge of changes in mortality and hospitalizations due to AD over time is critical.
